# Dr. Preston’s Case of Syphilis

**Published:** 1891-01

**Authors:** Mahlon Preston


### DR. PRESTON'S CASE OF SYPHILIS.

EDITORS OF THE HOMŒOPATHIC PHYSICIAN:

I was much gratified to receive the admonitions of my colleagues, Drs. Gee and Payne, which reached me through the pages of your September issue, concerning the report of a supposed case of syphilis which appeared in the August number of your journal, and which I had the honor to submit. General report, without doubt, will cordially receive these criticisms as emanating from a source quite qualified to speak *ex cathedra* on the subject, since the views there expressed coincide with opinions usually accepted as the true and recognized establishment of some of the widely-known experimentalists and writers on syphilitic ætiology. We find the medical profession of the present quite as prone as it has been in the past to yield support and credence to opinions which, though they may have originated from sources once official, have, of necessity, been superseded or modified by observations more recent and perfect. Yet certain parts of the older syphilitic doctrines still hold, by reason, I suppose, of reverence for the honored names who supported them. Thus much concerning the nature of syphilitic contagion formerly advanced still remains stubbornly contended for, against all modification whatever, but it is a pathological conception altogether, and one of little value in establishing the merits of the present case, which concerns more the phenomena observed and cited by those less tied to a regime already long past its climax. The diagnosis of my case is impugned on two counts:

1st. In that the period of inception, which was put at about ten days from the inoculation, is too short to admit of its being an instance of true chancre in any sense of the term.

2d. The nature of the ulcers was the chancroid variety, and of a consequence, innocuous, and void of the character capable of producing systemic contagion. Whether both critics desire to be so understood, is not quite certain, but Dr. Payne specifically declares this to be the ground of his objection.

Now, the moral integrity of a venereal subject can rarely be vouched for. One who involuntarily exhibits the *prima facie* evidences of moral debasement on his own person is never incapable of using any subterfuge to improve, however slightly, his moral standing, hence the ten-day limit may not be accurate in my case. It may very possibly have been longer, but I find by consulting a world-wide authority on syphilitic ætiology, that the primitive sore has been produced in a period of ten days. And if this corroboration had not been at hand the ellipsis could readily have been supplied from my personal observations had the evidence been admissible. Therefore, if the Doctor will patiently review the literature he will not find me alone in my views of this fact. The second count against my diagnosis, which assumes the case to be simply one of chancroid, must suffer a material repulse from this decided overthrow of the first; and, further, the authority which refuses to admit the possibility of the appearance of the primitive sore in a period of less than three to six weeks, also contends that chancroid does not take on the phagadenic form, into which condition the ulcer in my case speedily lapsed and threatened serious and vital destruction by reason of it. Several years since I witnessed the destruction of the whole penis by very similar sores, and by a very similar process, for which ulcers I was totally unable to discover a remedy in time to cut short the progress, until after the greatest mischief was accomplished. Since that sadly-recalled occasion I am not a heavy stockholder in the supposed non-infectious character of what is more popularly termed chanroid, believing strongly that this form plows as deeply, and makes its furrow plain enough to satisfy the boldest skeptic, come whence he may, proclaim what he will.

I pretend no expert knowledge on the syphilitic question, but what a student may know I claim the privilege of being able to know. I have stored my mind with the knowledge of a few remedies whose indications have saved me from committing many serious blunders, preserved my self-respect, and secured for my patients such comforts, relief, and cure as allopathic practice and pathological reasoning forbade them to anticipate. I do not often write for the journals or report for societies, because I do not very highly estimate my talent as a writer, or claim for myself the highest ability as a prescriber by any means.

Dr. Gee propounds a question to me, to which I feel myself free to reply with positiveness and enthusiasm in the affirmative. The manner and occasion of the interrogatory, however, possesses so strongly the ring of bitter irony that I venture here to repeat his question: "Do you think you ever wholly removed a miasm?" The cynical contortions of the doctor's face can well be imagined at his here implied contempt for the ridiculous claim of any one to have accomplished this thing, since he evidently believes he has never advanced so far as this point himself. This fact may elucidate the scriptural idea that wisdom is sometimes witheld from the learned and great and yet vouchsafed unto babes. I cannot refrain from recalling to the doctor's mind that memorable interrogative response given to Nicodemus, "Art thou a ruler (teacher) in Israel (Homœopathy), and knowest (believest) not these things?"

Postscript.—Since the above was written, I have learned ot the death of Dr. Gee. It will not, however, do him any injustice, so I shall not revise it.

Mahlon Preston, M. D.

